# Circadian rhythms and neuroendocrine dysregulation in ADHD: Therapeutic insights from omega-3 fatty acids

**DOI:** 10.37796/2211-8039.1679

**Published:** 2025-12-01

**Authors:** Ayesha Zafar Iqbal, Patricia Marest Suwindi, Sunny Yin-Shan Chen, Lindsay Liang-Tien Cho, Kuan-Pin Su, Jane Pei-Chen Chang

**Affiliations:** a Graduate Institute of Nutrition, China Medical University, Taichung 404, Taiwan; b Mind-Body Interface Research Center (MBI-Lab), China Medical University Hospital, Taichung, Taiwan; c College of Medicine, China Medical University (CMU), Taichung, Taiwan; d Department of Education, China Medical University Hospital, Taichung, Taiwan; e Office of Research and Development, Asia University, Taichung, Taiwan; f An-Nan Hospital, China Medical University (CMU), Tainan, Taiwan; g Child Psychiatry Division, Department of Psychiatry, China Medical University Hospital, Taichung, Taiwan

**Keywords:** ADHD, Circadian rhythm, Cortisol, Melatonin, Omega-3 PUFAs

## Abstract

Attention-deficit hyperactivity disorder (ADHD) is a common neurodevelopmental condition often accompanied by circadian rhythm disturbances, particularly delayed sleep phase. These involve suprachiasmatic nucleus (SCN) dysregulation, altered melatonin secretion, and hypothalamic–pituitary–adrenal (HPA) axis activity, which may be exacerbated by artificial light exposure. Genetic studies further implicate circadian mechanisms, linking ADHD with polymorphisms in clock genes such as *PER* and *CLOCK*.

Nutraceuticals, particularly omega-3 polyunsaturated fatty acids (n-3 PUFAs), have been proposed as modulators of circadian rhythms. N-3 PUFAs are essential for brain health and may influence melatonin synthesis and sleep–wake regulation. Preclinical and clinical findings suggest that supplementation can improve cognitive and behavioral outcomes in ADHD, possibly through circadian pathways, though direct clinical evidence remains limited.

This review integrates findings on melatonin and cortisol dysregulation in ADHD and evaluates n-3 PUFAs as potential non-photic zeitgebers. N-3 PUFAs may modulate circadian clock genes in the SCN, restore rhythm synchronization, normalize melatonin secretion, stabilize HPA axis activity, and reduce systemic inflammation. Future research should focus on well-designed trials to clarify the circadian effects of n-3 supplementation in ADHD.

## 1. Introduction

Attention-deficit hyperactivity disorder (ADHD) is one of the most common neurodevelopmental disorders that has been a burden worldwide and can potentially impact individuals and their families across the lifespan [1], with the common symptoms of inattention, hyperactivity, and impulsiveness [1]. The overall prevalence of ADHD in children and adolescents was 8.0% which is predicted to be twice as high in boys (10%) compared to girls (5%) [2]. Approximately 7.5%ADHDindividuals across all age groups had a formal sleep disorder diagnosis, and 47.5% are on prescribed sleep medications [3]. Among these disorders, delayed circadian rhythm is a common and significant feature in adults with childhood-onset ADHD. Studies indicate that up to 75% of these individuals exhibit delayed dim-light melatonin onset (DLMO), occurring about 1.5 h later than in individuals without ADHD, suggesting a significant alteration in circadian timing linked to the condition [4].

Furthermore, an epidemiological, genetic, etiological, and functional overlap exists between delayed circadian rhythms and ADHD symptoms across the lifespan [5]. In addition, the firstline treatment for ADHD is methylphenidate (MPH), a norepinephrine and dopamine reuptake inhibitor [6], which has been linked to adverse effects on sleep, including changes in total sleep time, sleep patterns, efficiency, and longer delay of sleep onset, potentially exacerbating sleep issues in individuals with ADHD [6]. However, these findings remain inconclusive due to conflicting results across studies and the limited availability of data. Factors such as the specific type of medication, dosage, and administration schedule warrant further investigation, as there is speculation that the pharmacological agents used may influence the chronobiology of the brain, thereby affecting sleep quality. Conversely, it is also plausible that improvements in daytime functioning resulting from medication use may subsequently lead to enhanced sleep quality during the night [7].

Circadian rhythms regulate physiological and behavioral processes on a 24-h cycle, though the actual human cycle often exceeds 24 h, requiring daily resetting [8]. This rhythm is controlled by a molecular clock involving feedback loops of core clock genes, including Circadian locomotor output cycles kaput *(CLOCK)*, Brain and Muscle Arnt-like 1 *(BMAL1)*, Period *(PER)*, Cryptochrome *(CRY)*, nuclear receptor subfamily 1 group D member 1 *(REVERBα)*, and Retinoid-related orphan receptor alpha *(RORαA)*. *CLOCK* and *BMAL1* dimerize to initiate *PER* and *CRY* transcription, with the resulting *PER-CRY* complex inhibiting *CLOCK/BMAL1* activity, creating a negative feedback loop. *REV-ERB* and *ROR* further regulate *BMAL1* transcription, contributing to the precision of the circadian cycle. Post-translational modifications fine-tune protein stability and turnover. For example, casein kinase 1ε/δ (CK1ε/δ) phosphorylates *PER* and *CRY* to control nuclear entry and degradation, and F-box/LRR-repeat protein 3 (FBXL3) targets *CRY* for proteasomal degradation, thereby resetting the feedback loop [9]. *CLOCK* acetylates *BMAL1* to facilitate *CRY*-mediated repression, whereas sirtuin 1 (*SIRT1)* deacetylates *BMAL1* and *PER2* to promote their turnover [10]. Together, these transcriptional and post-translational mechanisms ensure robust circadian rhythms, and their disruption leads to sleep and metabolic disorders.

While these molecular feedback loops constitute the circadian rhythms, their synchronization to the external environment is orchestrated by the suprachiasmatic nucleus (SCN), the central pacemaker located in the hypothalamus. The SCN synchronizes peripheral clocks mainly through light input from intrinsically photosensitive retinal ganglion cells via the retinohypothalamic tract (RHT) [11]. Although other oscillators exist outside the SCN, its role as the master pacemaker is underscored by evidence that lesions to the SCN disrupt a majority of physiological, endocrine, and behavioral rhythms [12]. Each SCN nucleus contains about 10,000 neurons and is positioned bilaterally to the third ventricle, just above the optic chiasm.

It is well-positioned to receive visual input for light-dark cycle entrainment via the RHT [13]. The melanopsin system, involving intrinsically photosensitive retinal ganglion cells, primarily mediates light's effects on the SCN [11]. These ganglion cell axons form the RHT, transmitting photic data to the SCN through a monosynaptic glutamatergic projection that includes the neuropeptide Pituitary Adenylate Cyclase-Activating Polypeptide (PACAP) [11]. Neuronal firing patterns in the SCN are crucial, as blocking SCN neuronal activity leads to behavioral arrhythmicity, which is restored upon resumption of SCN neuronal activity [14]. The SCN activity rises during the day and falls at night, following the same pattern as the clock genes and showing how the body internal clock controls SCN signals [15].

On the other hand, modern lifestyles, including irregular eating patterns, travel, caffeine intake, noise pollution, and excessive artificial light, can disrupt circadian rhythms, increasing the risk of cardiovascular diseases, cancer, neurodegenerative disorders, and ADHD [16].

## 2. Circadian rhythm disturbances in ADHD

### 2.1. Genetic associations of circadian rhythm disturbances and ADHD

Genome-wide association studies investigating such variants further support the involvement of disrupted molecular clock function in ADHD pathophysiology. These studies have increasingly highlighted the role of circadian *CLOCK* genes in ADHD, with particular attention to polymorphisms in the *PER* gene that influence circadian timing and behavioral regulation. A genome-wide association study investigating the impact of genetic polymorphisms on the circadian pathway in children with ADHD found a nominal association between the *Period 1* (*PER1)* gene and ADHD risk [17]. However, no significant changes were observed in *Period 2* (*PER2*) gene expression in cultured human dermal fibroblasts from patients with ADHD [18].

The T-allele of the r*s1801260* single-nucleotide polymorphism (SNP) in the *CLOCK* gene has been associated with adult ADHD symptoms [19] and is also linked to evening partiality and delayed sleep timing; however, the results are inconsistent [20,21]. Although the exact functional impact of this SNP on *CLOCK* gene expression or activity is unclear, its location in the 3’ untranslated region suggests it may influence messenger ribonucleic acid (mRNA) stability, translation, and polyadenylation signalling [19]. In addition to circadian *CLOCK* gene polymorphisms, genetic variations in extracellular matrix degradation pathways have also been implicated in the vulnerability to major depressive disorder (MDD) [22]. Together, these data highlight converging molecular mechanisms ranging from circadian regulation to ECM remodelling that may underlie shared pathophysiology across neuropsychiatric disorders such as ADHD and depression.

Animal models with *CLOCK* gene knockouts, such as zebrafish *PER1b* and mouse *PER1*, display ADHD-like behaviors, including hyperactivity, impulsivity, and attention deficits, which provides further evidence of the connection between circadian dysfunction and ADHD. The circadian system is also believed to influence ADHD by regulating dopamine levels. A study found that zebrafish with a mutation in the *PER1b* gene exhibit behaviors resembling those of individuals with ADHD, such as hyperactivity, impulsivity, and attention deficits, along with lower dopamine levels. The circadian clock regulates dopamine synthesis and the development of dopaminergic neurons, and its disruption, as seen in *PER1* knock-out mice, results in reduced dopamine levels and ADHD like hyperactive and inattentive behaviors, highlighting a link between circadian rhythm dysregulation and attention deficits [23].

### 2.2. Treatment for circadian rhythm disturbances in ADHD

Both non-pharmacological and pharmacological interventions are available to improve sleep in individuals with ADHD, targeting sleep disturbances associated with both the disorder itself and the side effects of ADHD medications. [24]. Non-pharmacological interventions include weighted blankets (a device made of weight-adding materials to cover the body evenly) [25], light therapy, acupuncture, exogenous melatonin, and other nutraceuticals. A metaanalysis showed that melatonin reduces sleep onset latency in children and adolescents with ADHD and comorbid insomnia [26]. Therefore, investigations into melatonin as a treatment for sleep disorders in ADHD patients have shown promise in improving total sleep time, shortening sleep onset delay, and enhancing emotional and behavioural regulation. However, the evidence remains limited and heterogeneous [27,28]. Moreover, nutraceuticals such as omega-3 polyunsaturated fatty acids (n-3 PUFAs) regulate neurological, inflammatory, metabolic, and cardiovascular processes through clock gene modulation, supporting their role as non-photic zeitgebers and potential therapeutics for circadian disruption-related pathologies [29]. N-3 supplementation has also shown benefits in ADHD and preventive effects against recurrent MDD, suggesting trans-diagnostic value [30]. The current work aims to provide an inclusive review of the prevailing literature on n-3 PUFAs in regulating circadian rhythms in individuals with ADHD.

## 3. Shared mechanisms between ADHD and circadian rhythm disorders

Several shared mechanisms, including the dysregulation of melatonin and hypothalamic–pituitary–adrenal (HPA) axis, have been proposed to link the pathophysiology of ADHD and circadian rhythm disorders with ADHD, further suggesting a complex interplay of biological factors in the disorder.

### 3.1. Melatonin dysregulation

Melatonin dysregulation may serve as a potential shared mechanism between ADHD and circadian rhythm disorders. As a key hormone modulating SCN and the circadian clock, it primarily influences the sleep-wake cycle. It is synthesized in pinealocytes from tryptophan, production peaks around 12–3 AM, and is controlled by sympathetic input from the cervical ganglion under SCN influence via GABAergic mechanisms [31]. It plays a crucial role in aligning circadian rhythms with the light-dark cycle, affecting sleep, hormone secretion, immune response, and daily activity patterns. Moreover, melatoninergic agonists have been used to treat non-24-h sleep-wake disorder in blind individuals [32].

Melatonin levels in individuals with ADHD have been shown to differ from those of controls, but with inconsistent results (Table 1). One study reported higher daytime and nighttime urinary melatonin metabolite (6-OH MS) levels in ADHD individuals compared to controls [33]. At the same time, another found no difference in the daily profile between controls and ADHD [34]. Additionally, a pilot study observed a lower 24-h melatonin rhythm in ADHD cases compared to controls [35]. However, some studies reported no significant differences in night-time melatonin levels [36,37]. Another study found overall higher melatonin concentrations in ADHD patients compared to controls [38]. A more consistent finding in ADHD is the delayed DLMO (circadian phase marker) and delayed sleep and wake times, particularly in individuals with sleep onset insomnia [39]. This delay is a characteristic of the delayed sleep phase syndrome (DSPS), suggesting that sleep onset insomnia is a circadian rhythm disorder comorbid with ADHD. Notably, individuals with the inattentive subtype of ADHD exhibit fewer symptoms of sleep-onset insomnia compared to other subtypes and tend to have longer and more stable sleep patterns [40]. This aligns with reports that subjects with the inattentive subtype of ADHD are sleepier during the day and sleep longer, potentially due to melatonin dysregulation [41]. However, not all studies report delayed sleep onset timing in ADHD, indicating variability in sleep disturbances within this population [42].

Various preclinical and clinical studies have explored the impact of melatonin on sleep and behavioral parameters, particularly in individuals with ADHD and related conditions. In a zebrafish model, auriculasin treatment was found to reduce hyperactivity, increase melatonin and dopamine levels, and regulate the circadian clock gene *PER1b* as illustrated in Table 2 [43].

Clinical studies involving children and adults with ADHD, sleep-onset insomnia (SOI), and DSPS have consistently reported improvements in sleep parameters following melatonin supplementation [44]. Findings include reduced sleep latency, increased total sleep time, and earlier DLMO, with no significant adverse effects reported [45,46]. The outcomes of these clinical trials are summarized in Table 2, further supporting the therapeutic role of melatonin for managing comorbid sleep disturbances in ADHD.

### 3.2. Hypothalamic–pituitary–adrenal (HPA) axis dysregulation

Hypothalamic-pituitary-adrenal (HPA) axis dysregulation has been reported in both circadian rhythm disorders and ADHD. The rhythmic output signals of the circadian system, originating from the SCN and areas influenced by the SCN, are crucial for synchronizing peripheral oscillators [47]. Cortisol, a key adrenal glucocorticoid stress hormone in the HPA axis, is a significant output orchestrated by SCN via arginine vasopressin and corticotropin-releasing hormone (CRH) [48]. Cortisol follows the circadian rhythm, rising before morning awakening, peaking within an hour, and gradually declining throughout the day [49]. Dysfunction in the behavioral inhibition system, possibly due to abnormal HPA axis responses to stress, has been proposed as a factor contributing to ADHD [50].

Lower cortisol levels in response to stress are associated with ADHD traits such as maladaptive behaviour and poorer cognitive performance in children [51]. Although inattentive subtypes also display blunted cortisol responses [52], some studies suggest that low cortisol responsivity is specific to the ADHD-combined type in children [53]. Factors such as treatment, comorbidity, and study design may account for these discrepancies. Additionally, sex differences in cortisol response have been noted, with boys exhibiting higher early morning cortisol levels and girls exhibiting lower levels [54]. Studies specify a phase delay in cortisol rhythm relative to wake time, though post-stress cortisol profiles remain unchanged in adults with ADHD [55].

Studies have indicated that ADHD is associated with alterations in cortisol secretion patterns, including a reduced cortisol awakening response (CAR) [56,57], lower overall cortisol levels compared to controls [58,59], and disruptions in cortisol rhythmicity, such as delayed acrophase (peak secretion time) [55] (Table 3). Some studies also reported increased cumulative diurnal cortisol and elevated morning and afternoon cortisol levels in ADHD individuals [60]. Additionally, variations in key circadian rhythm markers were observed, including lower *BMAL1* and *PER2* rhythms, reduced melatonin amplitude, and shortened actigraphic circadian periods in ADHD patients [55].

### 3.3. N-3 PUFAs deficiency

Another possible shared link between ADHD and circadian rhythm disorders is n-3 PUFAS deficiency. Literature supported the role of n-3 PUFAs in behavioral changes induced by inflammation in high risk groups such as children, pregnant women and patients with metabolic disorders [61]. Moreover, decreased brain-derived neurotrophic factor (BDNF) and increased pro-inflammatory cytokines such as Interleukin-6 and hsCRP have been shown in children with ADHD [59], while n-3 PUFAS have been shown to increase the expression of BDNF, tropomyosin receptor kinase B (TrkB), and cAMP response-element binding protein (CREB), which contribute to behavior and cognitive development and decrease the pro-inflammatory cytokines, including interleukin-1 (IL-1), interleukin-6 (IL-6), and tumour necrosis factor-alpha (TNF-α) in an in vitro study [62]. Previous studies demonstrated that ADHD children and adults with low n-3 index indicate more severe symptoms clinically (e.g., inattention and hyperactivity-impulsivity) [63] and physically, such as skin problems and dry eyes [64,65]. In response to this evidence, mounting studies of ADHD intervention by n-3 PUFAs supplementation, primarily with the high dosage of eicosapentaenoic acid (EPA) compared with docosahexaenoic acid (DHA), showed significant improvements in clinical symptoms [66] and cognitive performance in ADHD children and adolescents, in which particularly those with low endogenous levels of n-3 PUFAs [67].

Emerging research also highlights a potential role for n-3 PUFAs as circadian rhythm synchronizers. For instance, diets deficient in n-3 PUFAs have been shown to disrupt sleep patterns by affecting melatonin secretion and circadian clock gene expression [68]. Additionally, findings suggest a correlative association between sleep-wake cycles and fatty acid metabolism, as sleep deprivation has been linked to altered ketone body concentrations [69]. In vitro studies further demonstrate that palmitate can upregulate *BMALI*, a core circadian gene, but this effect is moderated in the presence of DHA, indicating that n-3 PUFAs may counteract the circadiandisrupting effects of saturated fats [70].

Based on the effects of n-3 fatty acids on the inflammatory process and cognition, which are regulated by the molecular clock, n-3 fatty acids may serve as circadian rhythm synchronizers. Various animal and human studies indicate that dietary fatty acids influence circadian rhythms. An experimental study conducted on Syrian Hamsters suggested that a diet deficient in n-3 PUFA disturbed nocturnal sleep, affecting the melatonin rhythm and also circadian clock functions [68]. Similarly, a large cohort study in type 2 diabetic patients described that marine n-3 PUFA consumption (EPA and DHA) was linked to reduced sleep impairment progression, mechanistically connected with increased central circadian regulation through upregulation of fundamental clock genes (*CLOCK, BMAL1, PER2*) and *RORα-*mediated *BMAL1* nuclear translocation, with restoration of hypothalamic clock oscillations [71].

## 4. N-3 PUFAs in improving circadian rhythm via melatonin and HPA axis in ADHD

N-3 PUFAs may be able to improve circadian rhythm via modulation of melatonin output and HPA axis function. A study demonstrated that an n-3-deficient diet in 2-month-old rats reduced pineal DHA levels and its proportion in the phospholipids, phosphatidylcholine, and phosphatidylethanolamine, leading to lower melatonin release [71]. Another study showed that rats on an n-3-deficient diet exhibited lower nocturnal urinary excretion of 6sulfatoxymelatonin (aMT6), a melatonin metabolite, a condition that was reversible with DHA supplementation [72]. Lavialle et al. further demonstrated that an n-3-deficient diet resulted in a higher arachidonic acid (AA)/DHA ratio and reduced nocturnal melatonin peaks in Syrian hamsters, accompanied by increased striatal dopamine levels and hyper-locomotion. These alterations were linked to decreased levels of 12-Hydroxyeicosatetraenoic acid (12-HETE), which is a signaling molecule that is derived from PUFAs, and the crucial role of DHA in maintaining neuronal membrane integrity, affecting the function of enzymes, receptors, and dopamine transporters [68].

The effects of dietary n-3 fatty acids on behavior and learning in 15-month-old SenescenceAccelerated Mouse Resistant 1 (SAMR1) strain mice were studied. Mice fed a perilla oil diet displayed lower incorrect responses in learning tests and showed increased n-3 PUFAs in brain phospholipids compared to those fed a safflower oil (n-3-deficient) diet. Despite higher overall activity in the n-3-deficient group, both diet groups maintained similar circadian activity patterns. This indicates that the n-6/n-3 ratio in the diet significantly affects brain lipid composition, learning ability, and activity levels [73].

HPA-axis activity also has a modulating effect on fatty acid metabolism. Cortisol plays a role in mobilizing, lysis, oxidation, and synthesis of fatty acids [74]. For example, Cortisol has been shown to suppress the activity of Δ5-and Δ6-desaturase, key enzymes responsible for introducing double bonds into fatty acid chains, thereby affecting the synthesis of PUFAs [75]. This inhibition seems to have differential effects on specific fatty acids, in such a way that high cortisol concentrations are associated with a decrease in n-3 PUFA concentrations [76]. Conversely, fatty acids themselves can influence HPA-axis function. Supplementation with long-chain n-3 PUFAs, such as EPA, has been found to reduce cortisol levels in both animal models and human studies [68,77]. Additionally, inadequate maternal intake of n-3 PUFAs during early development has been linked to increased HPA-axis activity in rodent offspring [78].

N-3 supplementation appears to alter HPA-axis regulation through multiple mechanisms. It increases circulating levels of EPA and DHA while reducing AA concentrations, potentially modulating stress responses in three key ways. First, the structural properties of fatty acids influence glucocorticoid receptor function depending on their chain length and degree of unsaturation [79]. Second, EPA and AA modulate the function of pglycoprotein, which plays a role in cortisol transport across the blood-brain barrier [80]. Third, the ratio of AA to EPA affects the balance of pro- and antiinflammatory eicosanoids, which can, in turn, regulate HPAaxis activity by influencing CRH secretion and glucocorticoid receptor sensitivity [81].

N-3 PUFAs play a role in modulating circadian rhythms via melatonin secretion and cortisol levels, associated with impaired sleep quality and disrupted sleep-wake cycles [71,82]. Currently, there is no clinical study specifically focused on the role of n-3 in regulating circadian rhythms in ADHD individuals; however, an animal study conducted on n-3 PUFAsdeficient diet hamsters exhibited symptoms such as disturbed melatonin rhythm and chronic locomotor hyperactivity [68] (Table 4). Differences in circadian architecture, neuroendocrine regulation, and behavioral complexity between these models and humans mean that findings may not fully extrapolate to clinical populations. Moreover, there is a relative scarcity of high quality human trials directly examining the effects of n-3 supplementation on circadian outcomes in ADHD, underscoring the need for well-designed clinical studies to validate these preclinical mechanisms. Some research has explored the impact of n-3 on sleep disorders in patients with ADHD. A study investigated the effects of n-3 PUFA supplementation containing 400 mg EPA, 40 mg DHA, in addition to 60 mg of n-6, in combination with 80 mg of magnesium and 5 mg of zinc on ADHD symptoms and sleep-related problems [83]. The study concluded that n-3 PUFAs showed promising effects in reducing ADHD symptoms, but no significant improvement was observed in sleep problems. Another study was conducted on preterm toddlers to check the effect of n-3, 6, and 9 on behavior and sleep, utilizing a supplement containing 706 mg total n-3 fatty acids (338 mg EPA, 225 mg DHA), 280 mg total n-6 fatty acids (83 mg GLA), and 306 mg total n-9 fatty acids (oleic acid), identified the improvement in socioemotional outcomes, but no effect was observed on sleep and behavior aspects [84]. Similar results were observed in another study, in which 200 mg/day DHA and 200 mg/day AA supplementation had no overall impact on child sleep. However, male children and children whose caregivers had depressive symptomatology seemed to benefit from DHA + AA supplementation in exploratory subgroup analyses, and the beneficial effects continued for some months following the end of supplementation [85]. Although, dietary n-3 PUFAs may improve cognitive regulation, behavior, sleep, and daytime functioning in ADHD, but findings remain inconsistent, largely due to methodological heterogeneity rather than lack of efficacy. Variability arises from dosage and EPA: DHA ratios, as many trials use modest doses (<1 g/day) that may be insufficient to correct deficiencies or affect circadian regulation. Higher EPA content appears more effective for core ADHD symptoms, whereas DHA is more directly linked to melatonin synthesis, membrane stability, and circadian gene modulation; thus, trials emphasizing one fatty acid may produce divergent outcomes. Intervention duration also varies, with shorter trials potentially underestimating benefits that require sustained neuronal incorporation of long-chain n-3 PUFAs. Finally, adjunctive treatments (e.g., magnesium, zinc, stimulants) complicate interpretation by obscuring the specific contribution of n-3s.

Circadian and neuroendocrine disturbances in ADHD represent an interconnected cascade rather than isolated events. N-3 PUFAs provide a modulatory link, influencing circadian timing, stress responses, and inflammation. A central premise is that n-3 PUFAs act on the master circadian clock in the SCN by regulating core clock genes such as *BMAL1* and *CLOCK*, thereby restoring synchronized rhythms. This normalization extends to downstream neuroendocrine and inflammatory pathways.

**Fig. 1:**
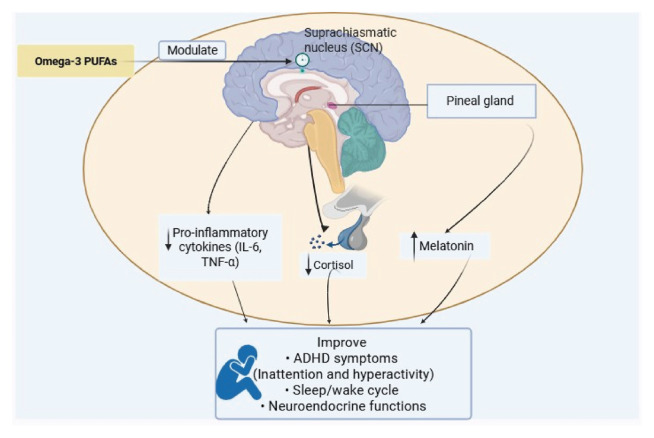
Summary of the potential role of omega-3 PUFAs in regulating circadian rhythm HPA-axis and ADHD symptomology. N-3 PUFAs modulate the master circadian clock (increase melatonin levels), neuroendocrine function (decrease cortisol levels), and inflammatory pathways (decrease pro-inflammatory cytokines levels such as IL-6 and TNF-α) thereby improving behavioral symptoms, sleep-wake cycle, and the neuroendocrine functions in ADHD. Note: ADHD: Attention deficit hyperactivity disorder, IL-6: Interleukin6, N-3 PUFAs: Omega-3 polyunsaturated fatty acids, SCN: Suprachiasmatic Nucleus, TNF-α: Tumor necrosis factor-α.

As the SCN regulates melatonin, n-3 deficiency has been associated with reduced melatonin rhythm and nocturnal sleep disturbances, whereas supplementation restores secretion and supports timely sleep. Similarly, the HPA axis and its cortisol rhythms are under circadian control. By dampening inflammation and lowering cytokines such as IL-6 and TNF-α, n-3 PUFAs indirectly stabilize cortisol release, often disrupted in ADHD. As summarized in Fig. 1, n-3 PUFAs act as central regulators, modulating circadian gene expression and aligning neuroendocrine and inflammatory outputs. This integrated mechanism addresses multiple facets of ADHD pathophysiology, highlighting the potential of n-3 supplementation as a holistic therapeutic strategy.

## 5. Conclusion

Sleep and circadian rhythm disruptions are known to exacerbate ADHD symptoms, highlighting the need for targeted clinical interventions. Emerging evidence indicates that n-3 PUFAs may represent a safe and well-tolerated therapeutic strategy for managing both ADHD symptoms and circadian rhythm disturbances. This review integrates ADHD, circadian mechanisms and n-3 PUFAs to propose a novel framework in which n-3 PUFAs offer a mechanistic bridge between metabolic, immune and chronobiological regulation in ADHD However, the lack of clinical trials directly assessing circadian endpoints in ADHD patients receiving n-3 supplementation remains a critical gap. Future studies should prioritize well designed clinical trials to evaluate the specific effects of n-3 supplementation on circadian rhythm disorders in individuals with ADHD, with a focus on determining the optimal dosage, timing, and formulation to maximize therapeutic outcomes.

## Figures and Tables

**Table 1:** Melatonin levels in ADHD.

Study (year)	Study Design	Participants (n)	Age (range or mean + SD) (y)	Sample type	Outcomes	

					ADHD	Control
Avcil et al., 2021 [38]	Case-control	ADHD: 103 C: 73	9.62 ± 1.17	Serum	Melatonin levels: 2.02 ± 0.74 pmol/L	Melatonin levels: 1.42 ± 0.44 pmol/L
Büber et al., 2016 [33]	Case-control	ADHD: 27 C: 28	9.95 ± 2.74	Urine	6-OH MS daytime: 15.4 (8.9–24.8) ng/mL; 6-OH MS night-time: 102.9 (65.3–197.7) ng/mL; 6-OH MS 24-h: 54.1 (34.6–83.9) ng/mL	6-OH MS daytime: 6.9 (2.5–15.9) ng/mL; 6-OH MS night-time: 61.5 (37.2–114.4) ng/mL; 6-OH MS 24-h: 27.3 (14.3–48.9) ng/mL
Cubero-Millán et al., 2014 [86]	Prospective, quasiexperimental, open-label clinical study	ADHD: 104 ADHD + Dep: 32	9.61 ± 2.54	Serum	Morning melatonin: (ADHD) 22.59 ± 11.97 pg/mL; (ADHD + depression) 22.13 ± 20.61 pg/mL; Night melatonin: (ADHD) 10.7 ± 8.91 pg/mL; (ADHD + depression) 12.35 ± 14.35 pg/mL	–
Dück et al., 2022 [35]	Pilot study Case-control	ADHD: 12 C: 11	9.67	Saliva	24 h rhythm for melatonin: 36.22%	24 h rhythm for melatonin: 53.50%
Molina-Carballo et al., 2013 [34]	Prospective, quasiexperimental, open-label clinical study	ADHD: 136 C: 42	9.70 ± 2.55	Morning melatonin: serum; evening melatonin: urine	Morning melatonin: 28.31 ± 25.08 pg/mL; Evening melatonin: 16.9 ± 19.42 pg/mL	Morning melatonin: 27.0 ± 18.94 pg/mL; evening melatonin: 14.6 ± 7.88 pg/ml
Nováková et al., 2011 [36]	Case-control	ADHD: 34 C: 43	6–12	Saliva	Night time melatonin: 43.2 ± 4.0 pg/mL	Night-time melatonin: 43.0 ± 3.7 pg/mL
Paclt et al., 2011 [37]	Case-control	ADHD: 34 C: 43 Anxiety: 11	6–12	Saliva	Melatonin levels (no significant difference)	

Abbreviations: ADHD: Attention Deficit Hyperactivity Disorder; C: Control; Dep: Depression; 6-OH MS: 6-Hydroxymelatonin Sulfate.

L: Litre; mL: Millilitre; N: Number; ng: Nano Gram; pg: Pico Gram, pmoL: Picomoles; SD: Standard Deviation; y: Years.

**Table 2:** Melatonin treatment in ADHD pre-clinical studies and clinical studies.

Pre-clinical study							

Study (year)	Animal model	Animals (n)	Treatment (n)	Behavioral parameters	Primary outcomes		
Wang et al., 2018 [43]	Zebrafish	2 Wild-type (WT) zebrafish: 24 *Period 1b* −/− zebrafish mutants: 24	1) WT (control): 12 2) WT + PI5 (incubated in 20 nmol auriculasin): 12 3) *Period 1b* mutants (control):12 4) p + PI5 group (incubated in 20 nmol auriculasin):12	Locomotor activity assays	↓ hyperactivity in the p + PI5 group, ↑ melatonin, and dopamine content, ↓ mao expression, Auriculasin regulates the circadian clock gene *Per1b*		

**Clinical studies**							

**Study (year)**	**Study Design**	**Participants**	**Age (range or Mean + SD) (y)**	**Intervention**	**Sample type**	**Primary outcomes**	**Other outcome**

Ayyash et al., 2015 [27]	Prospective observational	ID: 29, ADHD: 7, ASD: 9	6.3 ± 1.7	2.5–3 mg → 5 –6 mg → 9–10 mg melatonin vs placebo	Sleep diaries	↓ Awakening and sleep onset time, ↑ total sleep time	No AE
Checa-Ros et al., 2023 [87]	Open-label trial	ADHD: 27	9.67 ± 2.13	1 mg of fast-release exogenous melatonin (around 30 min before the usual bedtime)	Sleep diaries	↑ TST, no significant reduction of SOL, and small improvements in SE and WASO	No AE
Hoebert et al., 2009 [88]	RCT	ADHD + SOI: 105	9	(3 mg when body weight <40 kg [n = 44]; 6 mg when body weight >40 kg [n = 9]) vs placebo	Questionnaires	↓ sleep onset, ↑ total time asleep, ↓log item difficulty falling asleep, ↓sleep latency, 44.4 ± 67.9 min advance in DLMO, CBCL, TRF, TAC-QOL-P, Interference control (no significant effect)	No AE
Masi et al., 2019 [89]	Clinical trial	ADHD: 74	11.6	Mean dosage 1.85 ± 0.84 mg/d	CGI-I score	CGI-I score (↓ follow-up vs. baseline)	No AE
Mohammadi et al., 2012 [28]	RCT	Melatonin group: 29 Placebo group: 24	9.2 ± 1.74	Melatonin (3 mg when body weight <30 kg and 6 mg for >30 kg) + methylphenidate (Ritalin) (1 mg/kg) vs placebo combined with methylphenidate (1 mg/kg)	ADHD rating scale and SDSC questionnaire	↓ sleep latency and sleep disturbances, and ADHD scores	–
Tjio Pian Gi et al., 2003 [45]	Preliminary open-label trial	ADHD + Insomnia: 24	NI	3 mg melatonin	Clinical interview and psychological test	↓ Sleep onset time	–
Van Andel et al., 2021 [44]	RCT	ADHD + DSPS: 51	18–55	Sleep education: 3 weeks Melatonin (0.5 mg/day) Melatonin + BLT (10,000 lux for 30 min) Placebo (0.5 mg/day)	Saliva	DLMO (↑ Melatonin, Melatonin + BLT vs. Placebo: no effect) ADHD-RS-IV (↓ Melatonin vs. Melatonin + BLT: no effect)	No AE
Van Andel et al., 2022 [90]	RCT	ADHD + DSPS: 49	18–55	Sleep education: 3 weeks Melatonin (0.5 mg/day) Melatonin + BLT (10,000 lux for 30 min) Placebo (0.5 mg/day)	Actigraphy, self-reported sleep questionnaires, and a sleep diary	SDL SHQ ADHD-RS (no effect on sleep and ADHD symptoms)	–
Van Andel et al., 2024 [91]	RCT	ADHD + DSPS:37	18–53	Melatonin (0.5 mg/day) Melatonin + BLT (10,000 lux for 30 min) Placebo (0.5 mg/day)	Cortisol: saliva; leptin, insulin, ghrelin, IGF-1, and glucose: blood	Leptin and insulin (↓ Melatonin, melatonin + BLT, and placebo no effect)	
Van Maanen et al., 2017 [46]	RCT	Ễxprimental group: 16, Comparison group: 41	9.68	1 mg (maximum 5 mg) for 3 weeks, continued with half dose week	DLMO: Saliva Actigraphy and sleep diary	↑ DLMO, ↓ sleep-onset time	–
Weiss et al., 2006 [92]	Double-blind, placebo controlled, crossover trial	ADHD + Initial ínomnia: 27	10.29	10 days of sleep hygiene intervention, 30 days of 5 mg melatonin	Parentcompleted somnolog and actigraphy	↓ Initial insomnia	No AE
Van Maanen et al., 2017 [93]	RCT	Melatonin group: 26, Placebo group: 28, Light group: 30	10.0	Melatonin tablets (3 mg) or placebo. Light therapy consisted of daily bright blue-green light exposure (500 nm peak, 8000 lux for 30 min	Sleep dỉaies, Actigraphy, DLMO, CSRQ	↓ Sleep latency, ↑ DLMO, CSRQ: no effect	–

Note: ↑ Increase, ↓ Decrease.

Abbreviations: ADHD: Attention Deficit Hyperactivity Dísorder; AE: Adverse Events; ASD: Autism Spectrum Díorder; BLT: Bright Light Thẻapy; CBCL: Child Behaviour Checklist; CBT: Core Body Temperaturet; CGI: Clinical Global Impression; CSRQ: Chronic Sleep Reduction Questionnaire; DLMO: Dim Light Melatonin onset; DSPS: Delayed Sleep Phase Syndrome; ID: Intellectual Díability; IGF-1: Insulin-like Growth Factor-1; N: Number; RCT: Randomized Control Trial; SD: Standard Deviation; SDL: Sleep Diagnostic List; SDSC: Sleep Disturbance Scale for Children; SHQ: Sleep Hygiene Quetionnaire; SOI: Sleep onset Inomnia; SOL: Sleep onset Latency; TACQOL-P: TNO-AZL Questionnaire for Children’s Health-Related Quality of Life; TRF: Teacher Report Form; TST: Total Sleep Time; WASO = Awakenings after Sleep onset; WT: Wild Type; Y: Year.

**Table 3:** Cortisol levels in ADHD associated with circadian rhythm outcome.

Study (year)	Study Design	Participants (n)	Age (range or Mean + SD) (y)	Sample type	Primary outcomes	Other outcomes
Angeli et al., 2018 [57]	Case control	ADHD: 62 C: 40	8.4	Saliva	**CAR** (↓ ADHD vs. Control) α-amylase: (↓ ADHD vs. Control but NS)	
Baird et al., 2012 [55]	Case control	ADHD: 13 C: 19	31.3 32.3	Saliva	**Rhythm amplitude** (↑ ADHD vs. Control) **Actigraphic circadian period**: (↓ ADHD vs. Control) **Morning**–**Eveningness score:** (↓ ADHD vs. Control) **SOL:** (↑ ADHD vs. Control) **Sleep efficiency:** (↑ ADHD vs. Control) **Total sleep duration:** (↓ ADHD vs. Control) ***BMAL1* rhythm:** (↓ ADHD vs. Control) ***PER2* rhythm:** (↓ ADHD vs. Control)	**Cortisol Rhythm:** Acrophase (time of peak secretion) Delayed (~3 h after waking in ADHD) **Melatonin Rhythm:** **Amplitude:** (↓ ADHD vs. Control) **Circadian rhythmicity:** (↓ ADHD vs. Control but NS)
Berens et al., 2023 [60]	Cross sectional	Adolescents: 138	13.42	Saliva	↑ Cumulative diurnal cortisol (AUCg), morning cortisol, and afternoon cortisol in ADHD	No significant influence on cortisol level with the comorbidities (CD, anxiety, and depression)
Dück et al., 2022 [35]	Pilot study	ADHD: 12 C: 11	9.67	Saliva	24 h rhythmicity for cortisol and melatonin): ADHD: 49.63%; control: 66.31%,	–
Freitag et al., 2009 [56]	Case control	ADHD: 52 ADHD + ODD: 49 ADHD + CD: 22 C: 69	9.8	Saliva	CAR (ADHD without comorbidities vs. Control) ↑ AUC in ADHD + ODD	–
Fairchild et al., 2008 [94]	Case control study	EO-CD: 42 AO-CD: 28 C: 95	14–18 years	Saliva	***RCMAS*-2:** CON < EO **YPI:** CON < AO, EO **SES:** CON, AO < EO **Cortisol level:** no difference **CAR:** no difference **HR:** CON < AO, EO	–
Hirvikoski et al., 2009 [95]	Case control study	ADHD: 28 C: 28	ADHD = (19–50 years) Control = (19–54 years)	Saliva	**PSS:** (↑ Patients vs. ↓ Control) Cortisol Levels (Patients = Control)	–
Imeraj et al., 2012 [96]	Case control	ADHD: 33 C: 33	6–12 years	Saliva	**CBCL:** (↑ ADHD vs. Control) **CAR:** (↓ ADHD vs. Control but NS)	–
Isaksson et al., 2015 [58]	Case control	ADHD: 81 C: 88	ADHD = 12.5 years Control = 11.8 years	Saliva	**SNAP-IV:** (↑ ADHD vs. Control) **Cortisol levels:** (↓ ADHD vs. Control) **SNPs** = rs9470080 significantly associated with ADHD and low cortisol levels	–
Okabe et al., 2017 [97]	Longitudinal study	ADHD: 37 C:16	94	Saliva	**On awakening:** (↑ ADHD vs. Control) **30 min after awakening:** (↓ ADHD vs. Control) **CAR:** (↓ ADHD vs. Control)	**On awakening:** (↑ ADHD alone vs. ADHD + comorbidities) 3**0 min after awakening:** (↑ ADHD alone vs. ADHD + comorbidities) **CAR:** (↓ ADHD alone vs. ADHD + comorbidities: ASD, LD, ASD + LD, ASD + ODD, LD + ODD
Pesonen et al., 2011 [98]	Cross sectional	ADHD: 272	8 years	Saliva	No significant associations between ADHD symptoms and diurnal cortisol concentrations ADHD-I (↓ cortisol levels during or after TSST-C)	–
Ramos-Quiroga et al., 2016 [99]	Case control	ADHD-I: 46 ADHD-C: 64 C: 27	18–55	Saliva	**WURS** = (↑ ADHD vs. Control) **BDI** = (↑ ADHD vs. Control) **STAI-state** = (↑ ADHD vs. Control) **MCMI** = (↑ ADHD vs. Control) **BIS** = (↑ ADHD vs. Control) **CAR** = (↓ ADHD vs. Control but NS)	–
Chang et al., 2020 [59]	Case-control	ADHD: 98 Control: 21	6–18	Saliva	**Bedtime cortisol level** (↓ ADHD vs. Control)	–

Note: ↑ Increase, ↓ Decrease, < less than, > greater than.

Abbreviations: AC-CD: Adolescent onset Conduct; ADHD: Attention Deficit Hyperactivity Disorder; ADHD-C: Attention Deficit Hyperactivity Disorder-Combined; ADHD-I: Attention Deficit Hyperactivity Disorder-Inattentive; AO: Adolescent onset; AO-CD: Adolescent onset Conduct Disorder; ADHD-RS: Attention Deficit Hyperactivity Disorder-Rating Scale; ASD: Autism Spectrum Disorder; AUC: Area Under Curve; BMAL-1: Brain and Muscle Arnt-like 1; BDI: Beck Depression Inventory; BIS: Barratt Impulsiveness Scale; CAR: Cortisol Awakening Response; CBCL: Child Behavior Checklist; C: Control; CON: Control; EO: Early onset; EO-CD: Early onset-Conduct Disorder; HR: Heart Rate; PSS: Perceived Stress Scale; LD: Learning Disabilities: MCMI: Millon Clinical Multiaxial Inventory; N: Number NS: Non-significant; ODD: Oppositional Defiant Disorder; PER2: Period Circadian Protein Homolog 2; RCMAS-2:Revised Children’s Manifest Anxiety Scale; SD: Standard Deviation; SES: Socioeconomic Status; SNPs: Single Nucleotide Polymorphism; SNAP-IV: Swanson, Nolan, and Pelham Rating Scale; STAI: State-Trait Anxiety Inventory; TSST-C: Trier Social Stress Test for Children; vs: Versus; WURS: Wender Utah Rating Scale; Y: Years; YPI: Youth Psychopathic Inventory.

**Table 4:** Omega-3 PUFAs on ADHD reporting on sleeping outcomes.

Study (year)	Animal model	No. of Animals	Treatment	Behavioral parameters	Primary outcomes
Lavialle et al., 2008 [68]	Syrian hamster	24 hamsters (12 control diet; 12 (n-3) PUFA-deficient diet)	(n-3) PUFA-deficient diet: peanut oil (1200 mg LA/100 g) vs control diet (a mixture of peanut oil + rapeseed oil + 1200 mg LA + 200 mg LnA/100 g)	Locomotor activity (running wheels)	↓ melatonin rhythm, endogenous functioning of the circadian clock, ↑ and nocturnal sleep disturbances

Note: ↑ Increase, ↓ Decrease.

Abbreviations: ADHD: Attention Deficit Hyperactivity Disorder; LA: Linoleic Acid, LnA: α-Linolenic Acid, mg: Milligram, PUFA: Polyunsaturated Fatty Acid.
